# Supplementary material for: Enhancement of Neuroglial Extracellular Matrix Formation and Physiological Activity of Dopaminergic Neural Cocultures by Macromolecular Crowding
Source: Cells. 2022 Jul 6;11(14):2131. doi: 10.3390/cells11142131 (PMC9317039; doi:10.3390/cells11142131)
Supplement: Supplementary file 1 [file cells-11-02131-s001.zip › cells-1747523-supplementary.pdf]

cells

MDPI

Supplemetary Materials

Enhancement of Neuroglial Extracellular Matrix Formation and Physiological Activity of Dopaminergic Neural Cocultures by Macromolecular Crowding

Andy N. Vo [1,†], Srikanya Kundu [1,†], Caroline Strong [1], Olive Jung [1], Emily Lee [1], Min Jae Song [1], Molly E. Boutin [1], Michael Raghunath [2] and Marc Ferrer [1,*]

[1] National Center for Advancing Translational Sciences (NCATS), National Institutes of Health (NIH), 9800 Medical Center Drive, Rockville, MD 20850, USA; andy.vo@virginia.edu (A.N.V.); srikanya.kundu@nih.gov (S.K.); ces09f@my.fsu.edu (C.S.); olive.jung@nih.gov (O.J.); emily.lee@nih.gov (E.L.); minjae.song@nih.gov (M.J.S.); mollyboutin@gmail.com (M.E.B.)
[2] Department of Life Sciences and Facility Management, Institute for Chemistry and Biotechnology (ICBT), Zurich University of Applied Sciences (ZHAW),8820 Wädenswil, Switzerland; ragh@zhaw.ch
* Correspondence: marc.ferrer@nih.gov; Tel.: +1-(240)-515-4118
† These authors contributed equally to this work.

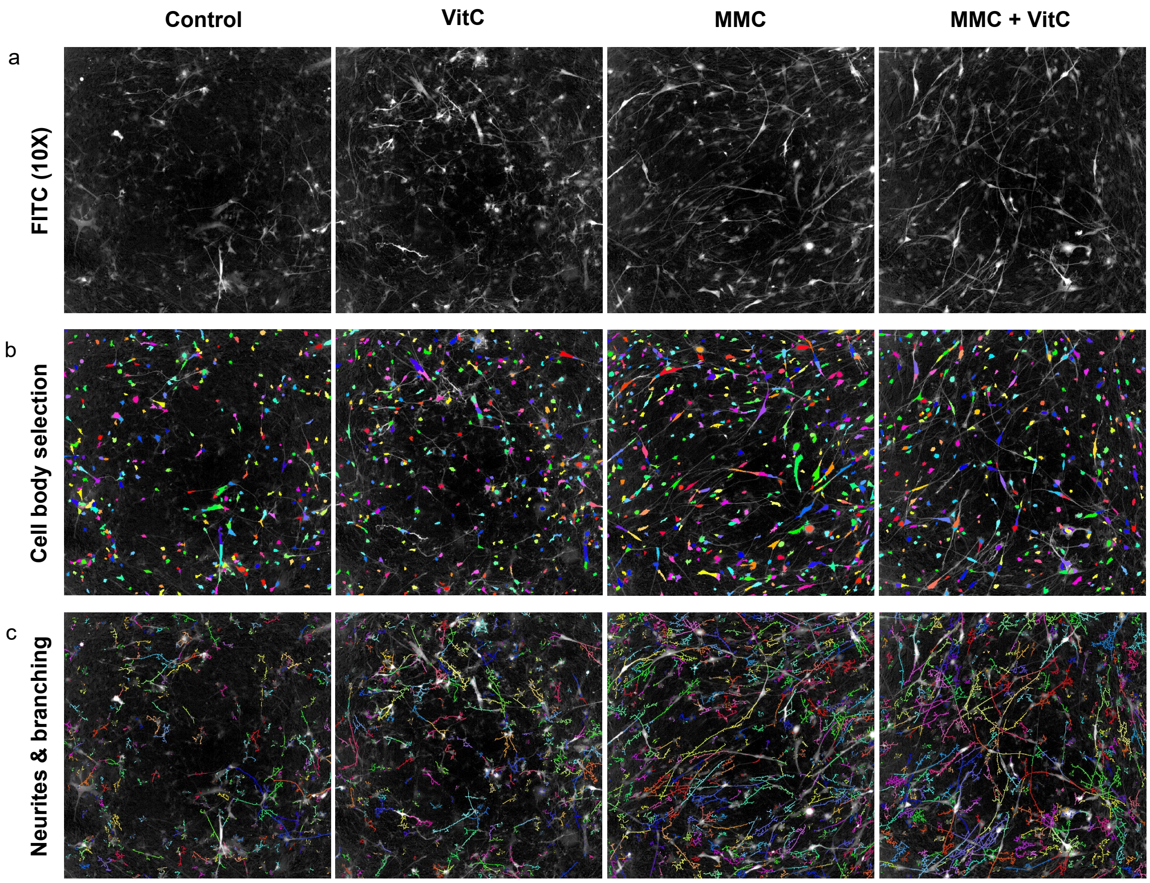

Figure S1. Image analysis for neurite extension and branching. (**a**) Representative images of GCaMP6f transfected cells from i) Control, ii) VitC, iii) MMC, iV) MMC+VitC groups taken under 10X water immersion confocal objective using FITC filter for neuronal morphology analysis. (**b**) The 'find cell' algorithmic script for image analysis in Columbus software (PerkinElmer) providing ROI of ≥20μm2 used to locate the neuronal cell body from each group. (**c**) CSIRO Neurites Analysis 2 method was applied to mark the length along with segment/branches of neurites originated from those preselected cell bodies. Scale bar: 50μm.

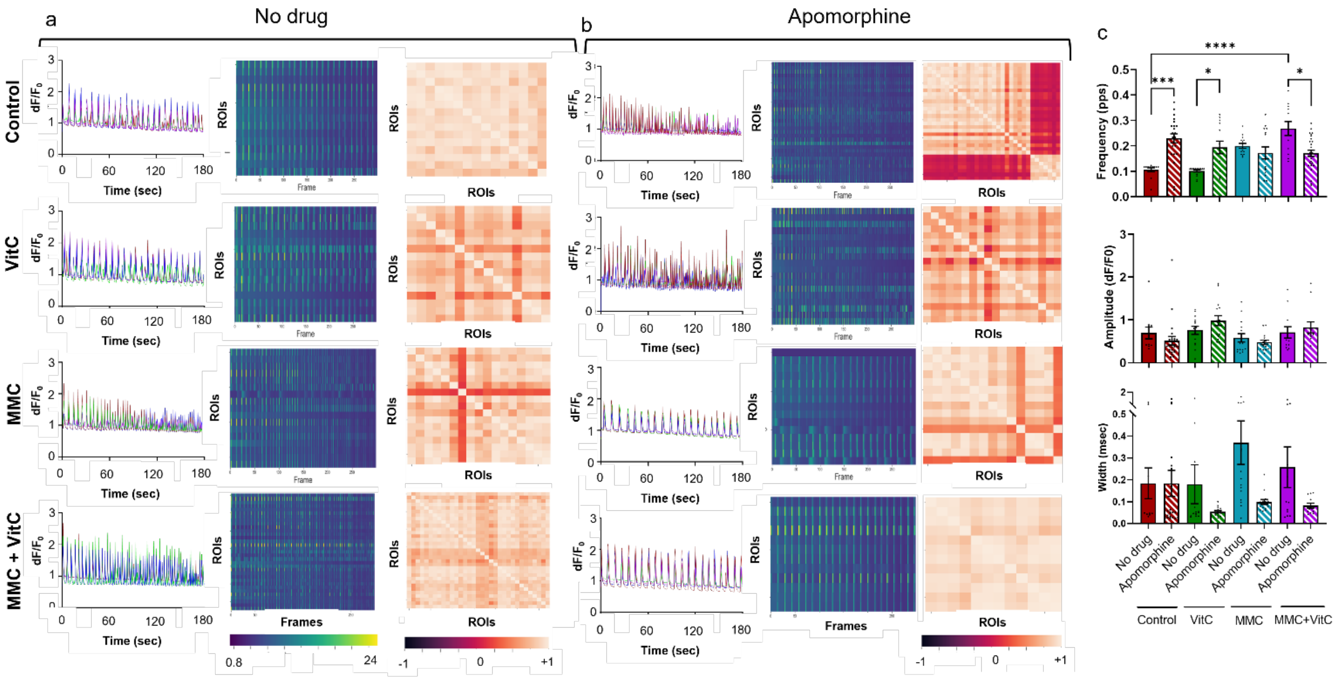

Figure S2. Effect of D2 receptor agonist (Apomorphine) on single neuronal calcium dynamics of iDopas+iAstros co-cultures under MMC treatment. (**a**) Analysis of basal single cell fluorescence calcium fluxes between MMC treatment groups, including example traces of calcium activity over time, activity combined heat map of over 30 neuros over time, and correlation heat map within cells. (**b**) Same as (a) after the Apomorphine treatment. (**c**) Quantitative calcium peak properties, peak frequency, peak amplitude, and peak width regulated with MMC treatment before and after Apomorphine treatment, along with the correlation score plot. Error bar: standard error of mean from 9 wells (n=3 wells/group, 3 biological replicates); Statistical significance: Two-way ANOVA, *$p < 0.05$, ** $p < 0.01$, *** $p < 0.001$, **** $p < 0.0001$.

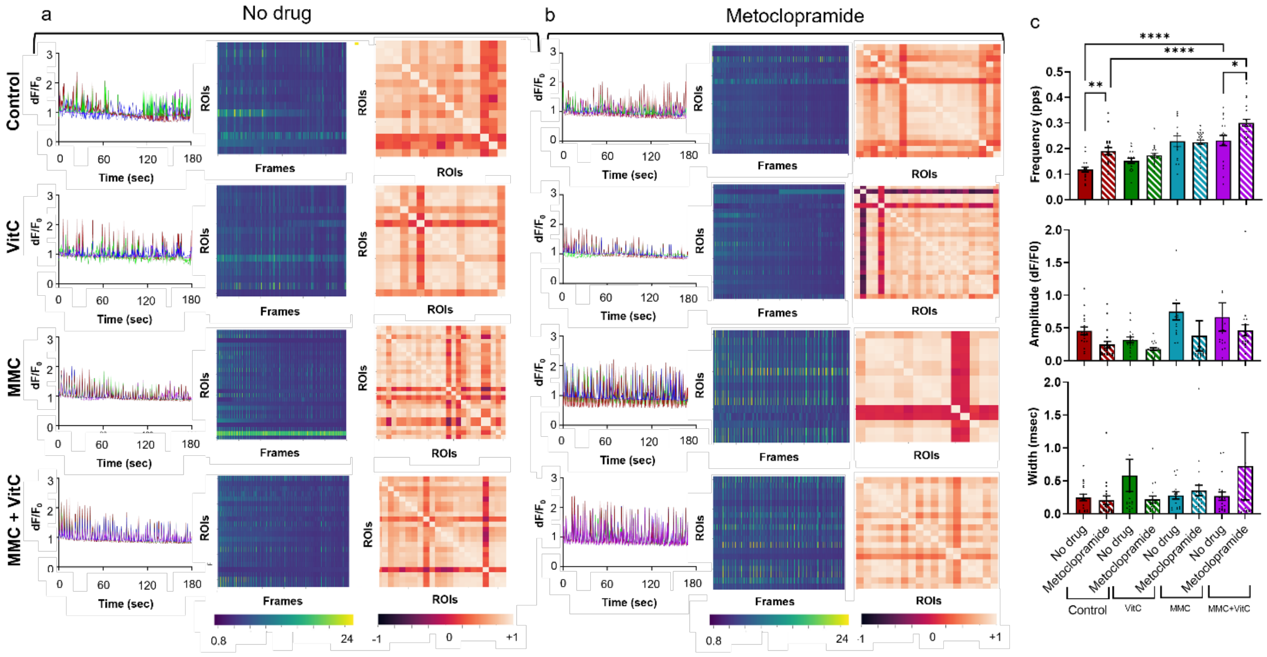

Figure S3. Effect of D2 receptor antagonist (Metoclopramide) on single neuronal calcium dynamics of iDopas+iAstros co-cultures under MMC treatment. (**a**) Analysis of basal single cell fluorescence calcium fluxes between MMC treatment groups, including example traces of calcium activity over time, activity combined heat map of over 30 neuros over time, and correlation heat map within cells. (**b**) Same as (a) after the Metoclopramide treatment. (**c**) Quantitative calcium peak properties, peak

frequency, peak amplitude, and peak width regulated with MMC treatment before and after Metoclopramide treatment, along with the correlation score plot. Error bar: standard error of mean from 9 wells (n=3 wells/group, 3 biological replicates); Statistical significance: Two-way ANOVA, * $p < 0.05$, ** $p < 0.01$, *** $p < 0.001$, **** $p < 0.0001$.

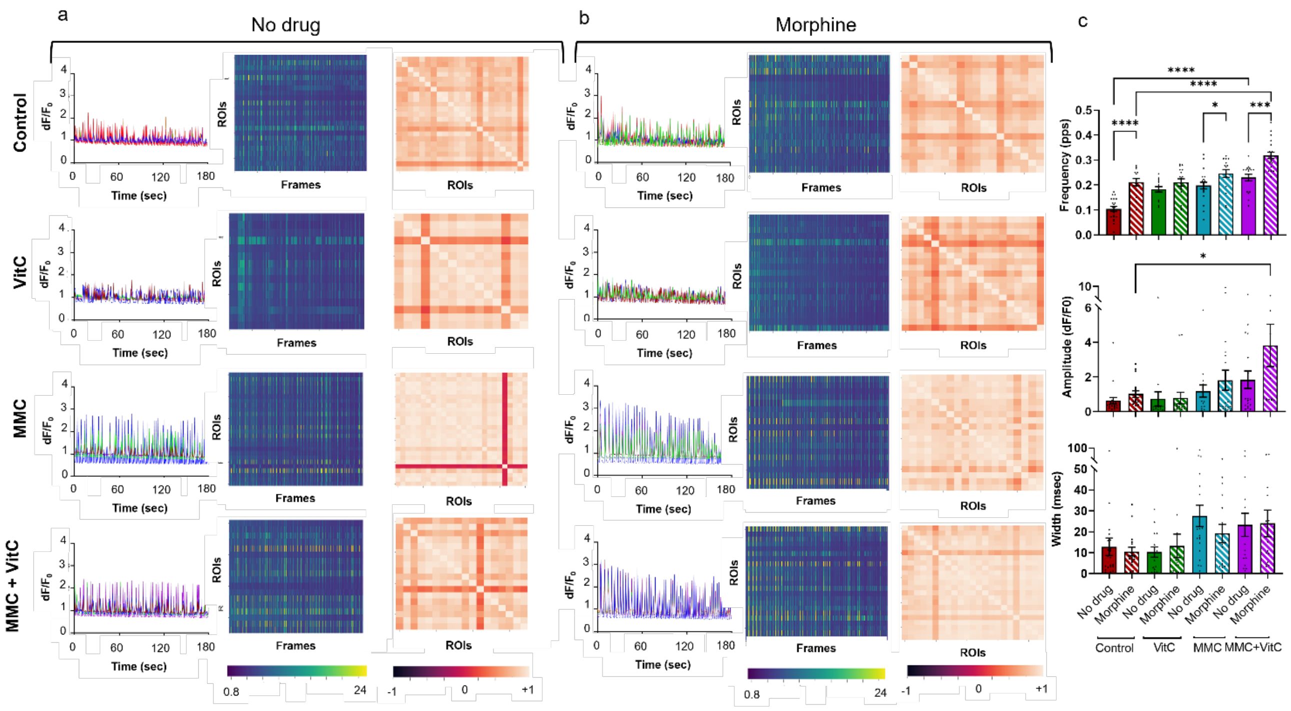

Figure S4: Effect of μ-opioid receptor agonist (Morphine) on single neuronal calcium dynamics of iDopas+iAstros co-cultures under MMC treatment. (**a**) Analysis of basal single cell fluorescence calcium fluxes between MMC treatment groups, including example traces of calcium activity over time, activity combined heat map of over 30 neuros over time, and correlation heat map within cells. (**b**) Same as (a) after the Morphine treatment. (**c**) Quantitative calcium peak properties, peak frequency, peak amplitude, and peak width regulated with MMC treatment before and after Morphine treatment, along with the correlation score plot. Error bar: standard error of mean from 9 wells (n=3 wells/group, 3 biological replicates); Statistical significance: Two-way ANOVA, * $p < 0.05$, ** $p < 0.01$, *** $p < 0.001$, **** $p < 0.0001$.

Table S1. Primary and secondary antibodies and dilutions.

Antibody Target (Clone)	Host & Clonality	Manufacturer & Catalog no.	Dilution
Collagen IV	Mouse monoclonal	Dako, M0785	1:200
Fibronectin	Rabbit polyclonal	Abcam, ab2413	1:200
GFAP	Rabbit polyclonal	Dako, Z0334	1:1000
Laminin (alpha1)	Rabbit polyclonal	Dako, Z0097	1:200
Microtubule-associated protein (MAP) 2	Mouse monoclonal	Sigma, M4403	1:200
Tyrosine hydroxylase (TH)	Rabbit polyclonal	EMD Millipore, AB152	1:1000
Goat anti-mouse 555 (secondary)		ThermoFisher, A28180	*1:500*
Goat anti-rabbit 647 (secondary)		ThermoFisher, A27040	*1:500*
Goat anti-chicken 488 (secondary)		Invitrogen, A32931	*1:500*
